# A Pilot Study to Examine the Correlation between Cognition and Blood Biomarkers in a Singapore Chinese Male Cohort with Type 2 Diabetes Mellitus

**DOI:** 10.1371/journal.pone.0096874

**Published:** 2014-05-09

**Authors:** Deborah Amanda Goh, Yanhong Dong, Wah Yean Lee, Way Inn Koay, Stephen Ziyang Tay, Danny Soon, Christopher Chen, Claire Frances Brittain, Stephen Loucian Lowe, Boon-Seng Wong

**Affiliations:** 1 Department of Physiology, Yong Loo Lin School of Medicine, National University of Singapore, Singapore, Singapore; 2 Department of Pharmacology, Yong Loo Lin School of Medicine, National University of Singapore, Singapore, Singapore; 3 Memory Ageing and Cognition Centre (MACC), National University Health System (NUHS), Singapore, Singapore; 4 Centre for Healthy Brain Ageing and Dementia Collaborative Research Centre, School of Psychiatry, The University of New South Wales, Sydney, New South Wales, Australia; 5 Lilly-NUS Centre for Clinical Pharmacology, Singapore, Singapore; 6 Lilly Erl Wood, Windlesham, Surrey, United Kingdom; University of Lancaster, United Kingdom

## Abstract

**Background:**

Diabetes is reported to be linked to poorer cognitive function. The purpose of this study is to examine (a) clinical correlation between cognitive function and the biochemical perturbations in T2DM, and (b) the impact of statin treatment on cognitive function in diabetic subjects.

**Methods:**

Forty Singaporean Chinese males with diabetes and twenty Singaporean Chinese males without diabetes were recruited for this study. Twenty-two of the diabetic subjects were on statin therapy and all subjects were non-demented. This was a 2-period non-interventional case-control study in which subjects were assessed for cognitive function in period 1 and blood samples taken over 2 periods, approximately 1 week apart. Blood was collected to determine the level of total cholesterol, high-density lipoprotein (HDL), low-density lipoprotein (LDL), triglycerides, glucose and insulin. Cognitive performance was measured by a neuropsychological battery covering domains of attention, language, verbal and visual memory, visuomotor speed and executive function. *Z-scores* were derived for each cognitive domain using the mean and standard deviations (*SD*s), and they were used to compare between (a) diabetic and non-diabetic groups, and (b) diabetic subjects with and without statin treatment. ANCOVAs with age, education, BMI, and the duration of diabetes as covariates were employed to examine differences in mean score of cognitive domains and subtests between the two groups.

**Results:**

Overall cognitive function was similar among diabetics and age matched non-diabetic controls. Among diabetic statin users, HDL, LDL and total cholesterol were negatively correlated with executive function, whereas peripheral insulin levels and insulin resistance were negatively associated with attention.

**Conclusion:**

Diabetic statin users were likely to have poorer performance in attention and executive function. Increasing levels of the peripheral biomarkers are likely to contribute to poorer cognitive performance.

## Introduction

The incidence of type 2 diabetes (T2DM) is rising globally [1], [2]. In Singapore, the number of diabetics grew 32% between 2005 and 2008 [3]. The social and economic cost of diabetes is high, due to the many problems that accompany diabetes, including vascular diseases and increased risk for cognitive impairment [4].

Insulin resistance is the fundamental defect [5] in T2DM [6]. While cognitive deficits have been reported in T2DM [7]–[9], very little is known about the origin and development of cognitive decline. Moreover, the effect of available T2DM treatments on the process of cognitive decline has not been examined.

Knowledge of cognitive deficits in T2DM may help in the management of the disease. Furthermore, if biomarkers can be identified and utilised at an early stage of this process, steps can be taken to slow the progression of cognitive decline into dementia. This will decrease caregivers and healthcare burden, especially in light of Singapore's ageing and increasingly obese population [3].

Insulin is an important modulator of growth and metabolic function [10]. However, knowledge on insulin function is derived from observations in the peripheral organ systems [5]. Although studies showed that insulin receptors (IRs) are abundantly expressed in the brain [4], [10], very little is known about the neuronal function of insulin.

Although insulin is known to enhance cognitive performance in non-T2DM [11], the connection between hyperinsulemia and cognitive impairment in T2DM is unclear [12], [13]. It is possible that the insulin resistant condition could prevent insulin from enhancing or preserving cognitive function. Since aberrant insulin signalling was widely observed in T2DM [14], [15], this perturbation could be contributing to cognitive impairment.

Individuals with T2DM are known to have increased cardiovascular disease (CVD) risk compared to non-T2DM [16]. Although cholesterol-lowering statin therapy has been shown to affect cognition in non-demented subjects [17], the effect of this therapy on the cognitive function in T2DM patients has not been investigated.

Therefore, the purpose of is study is to examine(a) the relationship between cognitive function and the biochemical perturbations in T2DM, and (b) the effect of statin treatment on cognitive function in diabetic subjects. The primary blood biomarkers measured were insulin, HDL, LDL, TG and cholesterol. In order to evaluate the validity of these analytes as biomarkers for clinical research, the inter- and intra- subject variability of each biomarker was also assessed.

## Methods

### Participants

This pilot study was approved by the National Healthcare Group (NHG) Domain Specific Review Board (DSRB) (protocol no 2011-00403). Written informed consent was obtained from all participants. All study procedures were carried out in accordance with the Declaration of Helsinki.

This was a 2-period non-interventional case-control study in which subjects were assessed for cognitive function in period 1 and blood samples taken over 2 periods, approximately 1 week apart.

Forty Chinese male T2DM subjects and twenty Chinese male subjects without T2DM (Table 1) were recruited from the community by Lilly-NUS Centre for Clinical Pharmacology. The diagnosis was confirmed by lab test results, including fasting blood glucose and HbA1c level, before they are classified under T2DM in the database. All the T2DM subjects were re-screened and the lab tests were done within a year to the time when they entered this study.

**Table 1:** Subjects profile (Note: Standard deviation in parentheses).

	Diabetics, *n* = 40	Non-diabetics, *n* = 20	Statistics for difference in subject profile
Demographics			
Age, years	60.6 (5.6)	60.2 (5.8)	*t*(58) = 0.31, *p* = 0.76
Education, years	13.1 (3.2)	12.0 (3.1)	*t*(58) = 1.28, *p* = 0.21
BMI, kg/m^2^	25.1 (3.3)	24.5 (2.1)	*t*(58) = 0.66, *p* = 0.51
Duration of Diabetes, years	10.6 (8.9)	0	*t*(39) = 7.50, *p*<0.0001
Global Cognitive Screening			
MMSE, /30	27.7 (1.5)	27.3 (1.3)	*t*(58) = 1.17, *p* = 0.25
MoCA, /30	26.3 (2.2)	26.4 (1.8)	*t*(58) = –0.18, *p* = 0.86

Twenty-two of these diabetic subjects were on statin therapy. None of the diabetics and non-diabetics subjects had a history of dementia based on medical examination. All the subjects have MMSE score of greater or equal to 26 (Table 1). For inclusion into the study, subjects were required to be male patients with type 2 diabetes mellitus (T2DM) as determined by the Investigator, between the ages of 50 and 85 years, and with a screening body mass index (BMI) of 18.5 and 35 kg/m^2^. Subjects were excluded if they were employees of NUS, NUHS, Lilly-NUS; had a significant history or presence of a medical condition that was capable of interfering with the interpretation of data or posed a risk to the subject participating in the study; showed evidence of significant active neuropsychiatric disease; had a history of drug or alcohol abuse; donated blood of 450 mL or more within 1 month of study entry or had an average weekly alcohol intake that exceeded 21 units per week (males up to age 65) and 14 units per week (males over 65).

The medications used by the subjects for their diabetes are Diamicron, Gibenclamide, Gliclazide, Glipizide, Januvia, Lantus, Levemir, Metformin, Mixtard, Novorapid flexpen, Sitagliptin.

### Blood Processing and Quantification of Biomarkers

Blood samples were taken from all enrolled subjects in each of the 2 periods, approximately 1 week apart. The objective of this repeated measure design was to assess potential biological variation of the blood biomarkers over 2 periods [18]. This would help sample size assessments in future studies utilising these biomarkers.

During each visit, 10 ml of venous blood was collected from each subject after overnight fasting. Blood samples drawn from the two visits were processed and analysed separately. The samples were centrifuged and separated into plasma, erythrocyte and haematocrit layer using Ficoll-Paque PLUS (BD Bioscience). Each blood fraction was stored separately in Eppendorf tubes at −80°C.

Biomarkers selected for quantification were plasma total cholesterol (C), high-density lipoprotein (HDL), low-density lipoproteins (LDL), triglycerides (TG), glucose, and insulin. Insulin resistance was calculated using the homeostasis model assessment of insulin resistance (HOMA-IR) [19].

Cholesterol, HDL, LDL and triglyceride levels were measured by colourimetry, using a Siemens Advia 2400. Insulin was measured by chemiluminescence, using a Siemens Advia Centaur. These tests were carried out on plasma samples. Glucose concentrations in plasma samples were measured using an Accu-check Aviva glucose meter.

### Cognitive and Clinical Measures

In this non-interventional case-control study, the cognitive functioning of all subjects were assessed in period 1. All 60 subjects underwent formal neuropsychological evaluation administered in English (n = 45) or in Chinese (n = 15) by trained research psychologists, blinded to the group status of the patients or controls.

The MMSE [20] and the Montreal Cognitive Assessment (MoCA) [21] were used as measures of global cognition. The formal neuropsychological battery adopted in this study was based on the National Institute of Neurological Disorders and Stroke - Canadian Stroke Network (NINDS-CSN) harmonization neurocognitive battery [22] (Table 2). In this study, this battery was modified for Singaporean subjects. These changes include (a) replacement of the Trail Making Test [23] with the Color Trails Test; (b) omission of the Wechsler Adult Intelligence Scale-III digit symbol; (c) omission of verbal fluency test, (d) added the Symbol Digit Modalities Test and (e) adding the digit span forward and backward.

**Table 2:** NINDS-CSN harmonization neurocognitive battery modified for Singaporeans [50].

Cognitive Domains	Subtests
Attention	Digit span forward and backwards
Executive Function	Colour Trail Test 1 and 2
Language	modified Boston Naming Test
Memory	Hopkins Verbal Learning Test (HVLT):
	Immediate recall
	30-min delayed recall and recognition
	Rey Complex Figure Test (RCFT):
	Immediate recall
	30-min delayed recall
Visuomotor Speed	Symbol Digit Modality Test
Visuospatial	RCFT copy

Neuropsychiatric symptoms that may co-exist with cognitive impairment were evaluated by the following assessments: (a) Neuropsychiatric Inventory, Questionnaire Version (NPI-Q) [24]; and (b) Geriatric Depression scale [25]. We have also administered the Bayer Activities of Daily Living Scale (B-ADL) [26] to evaluate daily functioning of the participants.

### Statistical Analysis

Student's t-test was carried out for the following pairs of groups to ensure that they are comparable on age, duration of education, BMI, and for the duration of diabetes: (i) diabetics & non-diabetics, and (ii) diabetic statin users & diabetic non-statin users. All significance levels reported were two-sided, with the standard alpha level of.05 (0.05) considered statistically significant.

Test-retest reliability estimates were calculated using intra-class correlation coefficients (ICC) [18], corresponding to a mixed-effects model in SAS 9.2 with visit (1 or 2), diabetic status (diabetic or non-diabetic) and statin usage (yes or no) fitted as fixed effects and subject as a random effect The ICC theoretical range from 0 to 1 is calculated as follows; ICC =  Between subject variance component/Total variance. An ICC≥0.70 is an acceptable level of test-retest reliability [27].

Cognitive performance was measured by a neuropsychological battery covering domains of attention, language, verbal and visual memory, visuomotor speed and executive function. *Z-scores* were derived for each cognitive domain using the mean and *SD*s of the (a) diabetic and non-diabetic groups, and (b) diabetic subjects with and without statin treatment. ANCOVAs using sample t-tests with age, education, BMI, and duration of diabetes as covariates were employed to examine differences in mean score.

Pearson correlational analysis was carried out to identify biomarkers whose levels significantly co-varied with cognitive performance as follows. Firstly, biomarkers (HDL, LDL, TG, cholesterol, insulin, HOMA-IR, glucose) were correlated with z scores of domain performance and global composite on the modified NINDS-CSN harmonization neurocognitive battery. Significant correlations (*p*<0.05) were reported. These correlations were then compared to corresponding biomarker-cognitive domain correlations using Fisher *r-*to-*z* transformation.

## Results

### Comparison of Diabetics and Non-Diabetic Controls

#### Population Characteristics of Diabetics and Non-diabetics

In this study population, the mean age of participants (diabetics and non-diabetics) was 60.5 years (S.D. = 5.6 years), average duration of formal education was 12.7 years (S.D. = 3.2 years) and BMI was 24.9 kg/m^2^ (S.D. = 2.9 kg/m^2^). Cardiovascular, cerebrovascular and psychiatric conditions, depression, alcohol abuse and substance abuse were either absent, or inactive. Hypertension was reported by 22 subjects (all diabetics) and hyperlipidemia was reported by 30 subjects (28 diabetics, 2 non-diabetics). Both the diabetics and non-diabetics did not differ significantly in age, education, BMI and global cognition screen – measured by the MMSE [20] and MoCA [21] (Table 1).

#### Cognition in Diabetics and Non-Diabetics

Performance on the modified NINDS-CSN Harmonization protocol did not differ significantly between both groups, with the exception of language (*p*<0.001) before (Table 3) and after (Table S1) controlling for age, education, BMI and duration of diabetes. However, the test for the language domain, the modified 15-item Boston Naming Test, has a ceiling effect – all non-diabetics had a full score of 15/15 and diabetics scored between 14/15 to 15/15.

**Table 3:** Table shows performance (*composite scores based on z-scores*) on cognitive domains of modified Harmonization protocol.

	Diabetics, *n* = 40	Non-diabetics, *n* = 20	Statistics for difference in performance
**Global Cognition**	−0.03 (0.54)	0.05 (0.54)	*t*(58) = −0.51, *p* = 0.61
**Memory**	0.02 (0.70)	−0.05 (0.75)	*t*(58) = 0.35, *p* = 0.73
Visual Memory	0.07 (0.82)	−0.14 (0.93)	*t*(58) = 0.90, *p* = 0.37
Verbal Memory	−0.02 (0.82)	0.05 (0.85)	*t*(58) = −0.32, *p* = 0.75
**Non-Memory**	0.01 (0.60)	−0.02 (0.62)	*t*(58) = 0.17, *p* = 0.86
Attention	0.02 (0.88)	−0.04 (0.67)	*t*(58) = 0.25, *p* = 0.81
Executive Function	−0.03 (0.91)	0.05 (0.94)	*t*(58) = −0.32, *p* = 0.75
Visuomotor Speed	0.01 (0.90)	−0.01 (1.20)	*t*(58) = 0.06, *p* = 0.96
Visuospatial Function	−0.02 (1.07)	0.04 (0.86)	*t*(58) = −0.21, *p* = 0.84
Language	−0.22 (1.17)	0.44 (0)	*t*(39) = −3.61, *p*<0.0001

Each value represents the mean (Standard Deviation).

#### Blood biomarkers in Diabetics and Non-Diabetics

**Figure 1:**
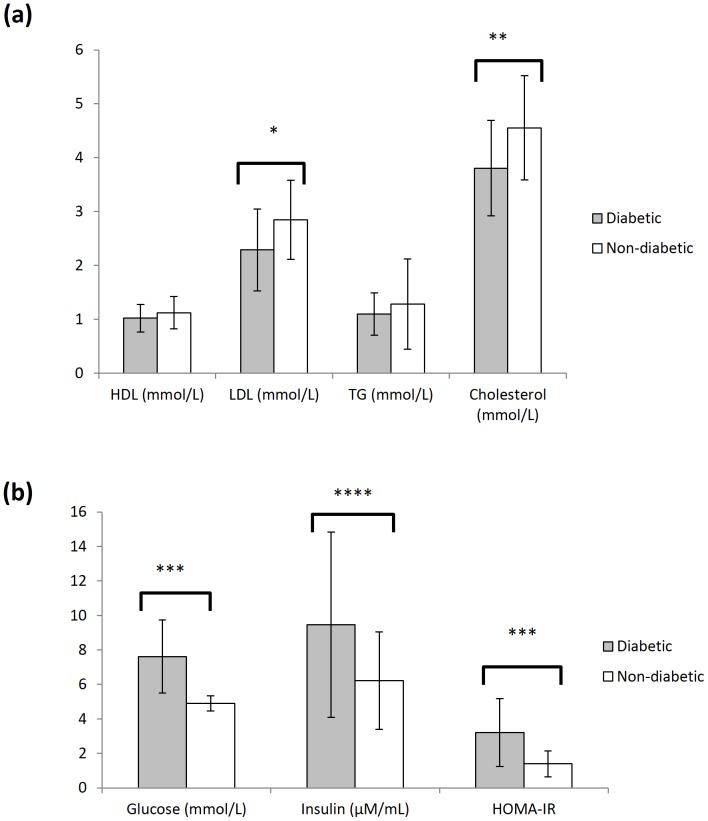
Analysis of blood biomarkers in study subjects. (A) The level of blood HDL, LDL, TG and cholesterol in diabetics (n = 40, grey) and non-diabetics (n = 20, white) subjects. (B) The level of blood glucose and insulin in diabetics (n = 40, grey) and non-diabetics (n = 20, white) subjects. HOMA-IR values are computed with the measured blood glucose and insulin levels using the formula given in the methods section [19]. Each value represents the mean ± SD of duplicate assays for individual samples (*p = 0.009; **p = 0.004; ***p<0.001; ****p = 0.014, using Student's t-test).

Diabetics had significantly lower LDL (Mean (M) = 2.29 mmol/L, SD = 0.76, *p* = 0.009) and total cholesterol (M = 3.81 mmol/L, SD = 0.89, *p* = 0.004) levels than non-diabetics (M = 2.85 mmol/L, SD = 0.73; M = 4.56 mmol/L, SD = 0.97, respectively) (Figure 1a), but significantly higher glucose (M = 7.62 mmol/L, SD = 2.13 versus M = 4.91 mmol/L, SD = 0.44, *p*<0.001), insulin (M = 9.46 µM/mL, SD = 5.37 versus M = 6.22 µM/mL, SD = 2.82, *p* = 0.014), and insulin resistance (HOMA-IR) (M = 3.20, SD = 1.97 versus M = 1.39, SD = 0.75, *p*<0.001) (Figure 1b).

#### Inter- and Intra-subject Variability of Biomarkers in Diabetics and Non-Diabetics

The four parameters tested in the blood lipid panel have above acceptable test-retest reliability across visits for both diabetics and non-diabetics (ICC = 0.86–0.96). The ICC was approximately equivalent between diabetics and non-diabetics for cholesterol, HDL and LDL (Table 4).

**Table 4:** Test-re-test reliability of blood biomarkers between 2 visits. (SD: Standard deviation; ICC: Intraclass Correlation Coefficient).

	Non-Diabetics(Mean)		Diabetics(Mean)		SD (intra subject)	SD (inter subject)	ICC Non- Diabetics, *n* = 20	ICC Diabetics, *n* = 40
	1^st^ Visit	2^nd^ Visit	1^st^ Visit	2^nd^ Visit				
***Blood Biomarkers***								
**Cholesterol (mmol/L)**	4.56	4.52	3.81	3.88	0.25	0.86	0.96	0.92
**HDL (mmol/L)**	1.13	1.88	1.02	1.02	0.12	0.26	0.92	0.93
**LDL (mmol/L)**	2.85	2.79	2.29	2.33	0.31	0.67	0.86	0.88
**TG (mmol/L)**	1.29	1.37	1.10	1.23	0.24	0.60	0.95	0.85
**Insulin (µU/L)**	6.22	7.55	9.46	10.58	3.67	5.82	0.39	0.68

The test-retest reliability between visits for insulin measurements was weaker although it was improved for diabetics (0.68) than for non-diabetics (0.39).

As the between-person variance is much greater than the within-person variance over the test-retest period [28], all four blood lipid panel tests can be deemed reliable.

It should be noted that the mixed model used in the statistical analyses assumes homogeneity of variances; this could not be confirmed for TG and insulin therefore results for these parameters should be interpreted with care.

The correlation analysis was conducted between cognitive performance assessed in Period 1 and the biochemical biomarkers measured in Period 1. Correlation analysis using blood biomarkers averaged between Period 1 and 2 showed very similar results.

### Comparison of Diabetic Statin Users and Diabetic Non-statin Users

#### Population Characteristics of Diabetic Statin Users and Diabetic Non-statin Users

Among the forty Singaporean Chinese males with diabetes, twenty-two of the diabetic subjects were on statin therapy. With reference to Table 5, diabetics statin users and diabetics non-statin users did not differ significantly in age, education, BMI, or duration of diabetes. The two groups also did not differ significantly in MoCA and MMSE.

**Table 5:** Profile of diabetics subjects with and without statins treatment. (Note: Standard deviation in parentheses).

	Statin Users, *n* = 22	Non-statin Users, *n* = 18	Statistics for difference in subject profile
Demographics			
Age, years	61.6 (5.7)	59.4 (5.3)	*t*(38) = 1.22, *p* = 0.23
Education, years	12.8 (3.1)	13.5 (3.4)	*t*(38) = −0.63, *p* = 0.53
BMI, kg/m^2^	25.8 (3.6)	24.1 (2.7)	*t*(38) = 1.67, *p* = 0.10
Duration of Diabetes, years	9.8 (7.7)	11.6 (10.4)	*t*(38) = −0.62, *p* = 0.54
Global Cognitive Screening			
MMSE, /30	28.1 (1.3)	27.3 (1.6)	*t*(38) = 1.70, *p* = 0.10
MoCA, /30	26.3 (2.2)	26.3 (2.2)	*t*(38) = 0.06, *p* = 0.95

#### Cognition in Diabetic Statin Users and Diabetic Non-statin Users

Performance on the modified NINDS-CSN Harmonization protocol did not differ significantly between both groups, before (Table 6) and after (Table S2) controlling for age, education, BMI and duration of diabetes.

**Table 6:** Table shows performance (composite scores based on *z­*scores) on cognitive domains of modified Harmonization protocol.

	Statin Users, *n* = 22	Non-statin Users, *n* = 18	Statistics for difference in performance
**Global Cognition**	−0.00 (0.55)	−0.05 (0.54)	*t*(38) = 0.30, *p* = 0.77
**Memory**	0.05 (0.65)	−0.01 (0.77)	*t*(38) = 0.23, *p* = 0.82
Visual Memory	0.05 (0.85)	0.09 (0.81)	*t*(38) = −0.16, *p* = 0.88
Verbal Memory	0.04 (0.73)	−0.11 (0.94)	*t*(38) = 0.56, *p* = 0.58
**Non-Memory**	0.02 (0.63)	0.00 (0.59)	*t*(38) = 0.13, *p* = 0.90
Attention	0.12 (0.92)	−0.10 (0.83)	*t*(38) = 0.80, *p* = 0.43
Executive	0.10 (0.98)	−0.19 (0.81)	*t*(38) = 1.01, *p* = 0.32
Visuomotor Speed	−0.08 (1.08)	0.10 (0.63)	*t*(38) = −0.63, *p* = 0.54
Visuospatial Function	0.13 (0.65)	−0.20 (1.44)	*t*(38) = 0.95, *p* = 0.35
Language	−0.16 (1.14)	−0.30 (1.23)	*t*(38) = 0.36, *p* = 0.72

Each value represents the mean (Standard Deviation).

#### Blood Biomarkers in Diabetic Statin Users and Diabetic Non-statin Users

**Figure 2:**
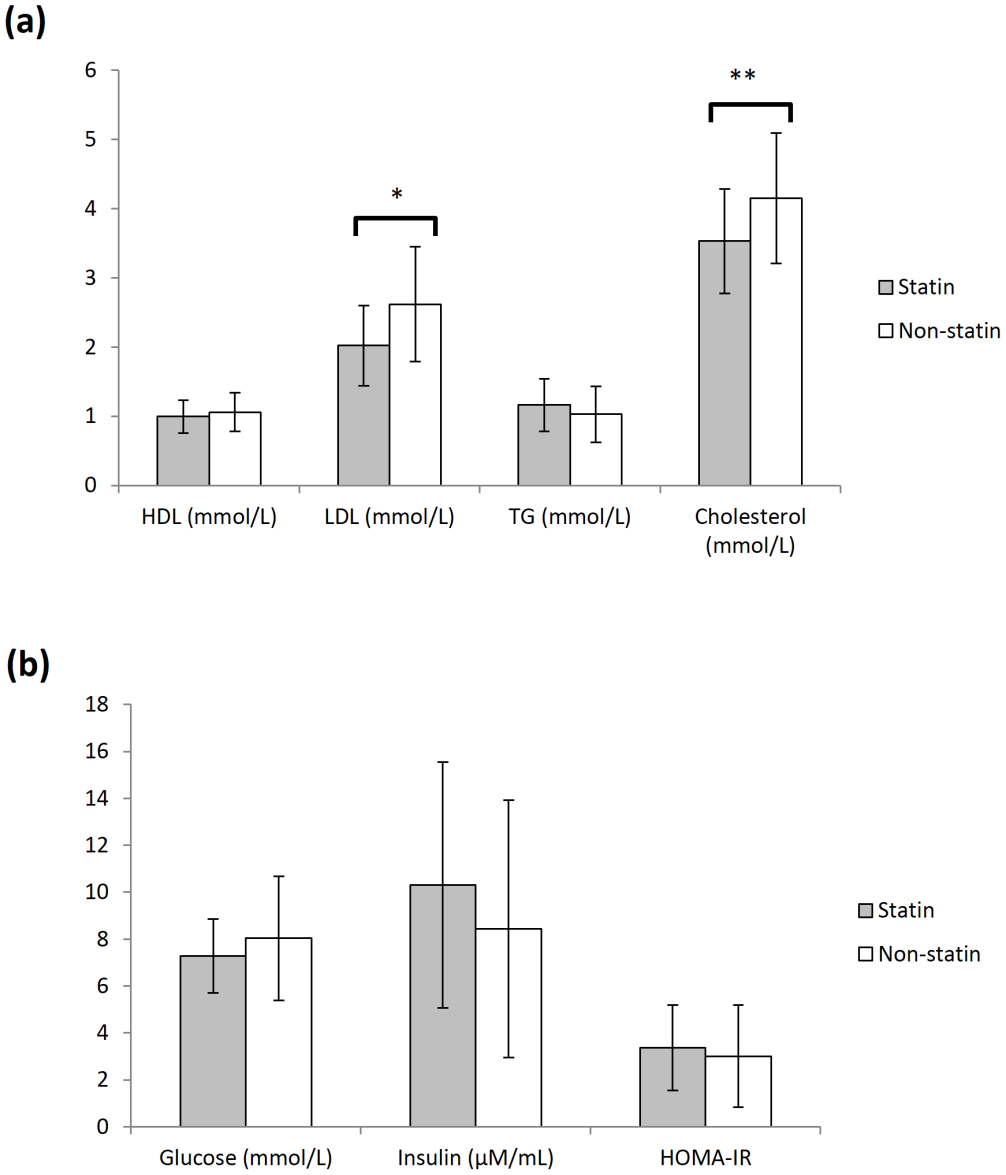
Analysis of blood biomarkers in diabetics subjects with and without statin treatment. (A) The level of blood HDL, LDL, TG and cholesterol in diabetics subjects with statin (n = 22, grey) and without statin (n = 18, white) treatment. (B) The level of blood glucose and insulin in diabetics subjects with statin (n = 22, grey) and without statin (n = 18, white) treatment. HOMA-IR values are computed with the measured blood glucose and insulin levels using the formula given in the methods section [19]. Each value represents the mean ± SD of duplicate assays for individual samples. (*p = 0.011; **p = 0.026, using Student's t-test).

Statin users had significantly lower LDL (M = 2.02 mmol/L, SD = 0.58 versus M = 2.62 mmol/L, SD = 0.83, *p* = 0.011) and total cholesterol (M = 3.53 mmol/L, SD = 0.75 versus M = 4.15 mmol/L, SD = 0.94, *p* = 0.026) levels (Figure 2a).

#### Correlation between Executive Function Task and HDL

Pearson correlational analysis was employed to identify biomarkers that were significantly co-varied with cognitive domain performance (Table 7).

**Table 7:** Table correlating the performance composite scores of specific cognitive domains with blood biomarkers using independent sample t-tests among statin users.

Biomarker	Domain	Statin Users, *n* = 22	*p*-value	Non-statin Users, *n* = 18	*p*-value
HDL	Executive Function	−0.655	0.001	0.418	0.084
LDL	Executive Function	−0.454	0.034	0.453	0.059
Cholesterol	Executive Function	−0.515	0.014	0.410	0.091
Insulin	Attention	−0.528	0.012	0.177	0.481
Insulin Resistance	Attention	−0.561	0.007	0.148	0.148

Correlations controlled for age, diabetic duration, BMI, years of education.

**Figure 3:**
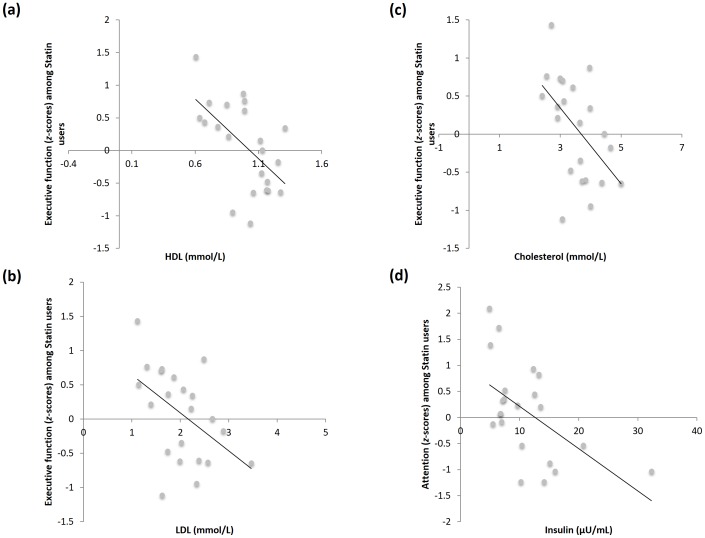
Pearson correlational analysis of cognitive function with blood biomarkers in diabetics subjects with statin treatment. Pearson correlational analysis was carried out to identify biomarkers whose levels significantly co-varied with cognitive performance in diabetic statin users. The blood biomarkers were correlated with z scores of domain performance and global composite and analyzed using Fisher r-to-z transformation. Significant correlation between (a) executive function with blood HDL, (b) executive function with blood LDL, (c) executive function with blood total cholesterol, and (d) attention performance with blood insulin levels among the diabetic statin users (n = 22; *p*<0.05 using Student's t-test).

In statin users those with higher HDL levels had better executive function (*r* = −0.655 *n* = 22, *p* = 0.001) (Figure 3a). However, the correlation between HDL and executive function task duration was not statistically significant within diabetic non-statin users (*r* = 0.418, *n* = 18, *p* = 0.084). The difference between these correlations (between diabetic statin users and diabetic non-statin users) was significant (*z* = 3.559, *p* <0.001).

#### Correlation between Executive Function Task and LDL

There was a no significant correlation between executive function task and LDL among diabetic non-statin users (*r* = 0.453, *n* = 18, *p* = 0.059) (Figure 3b). However, a significant negative correlation was observed between executive function task and LDL (*r* = −0.454 *n* = 22, *p* = 0.034) among the diabetic statin users. Moreover, the difference between these two correlations (between diabetic statin users and diabetic non-statin users) was significant (*z* = 2.832, *p* = 0.005). These results suggest that the correlation between the domain of executive function and LDL cholesterol levels are specific to diabetic statin users.

#### Correlation between Executive Function and Total Cholesterol

Among statin users, there was a significant negative correlation between executive function and total cholesterol levels (*r* = −0.515, *n* = 22, *p* = 0.014) (Figure 3c). However, correlation among diabetic non-statin users was non-significant (*r* = 0.410, *n* = 18, *p* = 0.091). Moreover, the difference between these two correlations (between diabetic statin users and diabetic non-statin users) was significant (*z* = 2.91, *p* = 0.004). Thus, the correlation between the domain of executive function and total cholesterol was found to be specific to diabetic statin users.

#### Correlation between Attention Scores and Peripheral Insulin

Performance on attention was found to be negatively correlated to peripheral insulin levels among statin users (*r* = −0.528, *n* = 22, *p* = 0.012) (Figure 3d). There was no significant insulin-attention correlation among diabetic non-statin users (*r* = 0.177, *n* = 18, *p = 0.*481). Moreover, there was a significant difference (z = 2.218, *p* = 0.027) between this correlation and the significant negative correlation among diabetic statin users. Therefore, this statistically significant correlational difference supported the finding that the negative insulin-attention correlation was specific to diabetic statin users.

#### Correlation between Attention Scores and Insulin Resistance

Among diabetics statin users, attention and insulin resistance were negatively correlated (*r* = −0.561, *n* = 22, *p* = 0.007) (Table 7). This negative correlation were non-significant among diabetics who are non-statin users (*r* = 0.148, *n* = 18, *p* = 0.557) (Table 7). The difference between these correlations was statistically significant (*z* = 2.268, *p* = 0.023). Thus, higher insulin resistance is associated with poorer attention task performance among diabetics, but only if they are statin-users.

## Discussion

Diabetes is reported to be linked to poorer cognitive function [7]–[9]. In this study however, we did not observe cognitive deficits in our local Chinese diabetic cohort. This could due to our younger community study subjects as compared to other studies [8], [9], [29]–[33].

Diabetic subjects are known to have increased cardiovascular disease (CVD) risk and are on statin treatment [16]. Cognitive problems caused by statin have been reported [34] as changes to cholesterol can affect cognition [35]. However, the cognitive performance of our diabetic subjects with and without statin treatment did not differ. It is possible that statin usage may predispose users to the development of memory disorders at an older age [36], [37].

The primary biochemical biomarkers used in the correlation analysis were insulin, HDL, LDL, triglyceride and cholesterol. An assessment of the inter-period variability of these biochemical biomarkers showed that they were reasonably consistent over 2 study periods and thus can be reliably used as biomarkers.

Our analysis shows that among the diabetic statin users, HDL, LDL and total cholesterol were negatively correlated with executive function. In non-diabetic older subjects, similar relationship between HDL, LDL and total cholesterol with cognitive function has been documented [38]–[41].

In contrast, we have observed a negative correlation between peripheral insulin level and attention, which is observed in diabetics who are statin users, but not in diabetics who are non-statin users. While similar deficits in attention and executive function were reported in studies documenting cognitive impairment in diabetics subjects [7]–[9], it is unclear if these subjects are on statin therapy.

Collectively, our findings suggest that with statin use, HDL, LDL and total cholesterol levels can predict performance on executive function; and both peripheral insulin and insulin resistance can predict performance on attention. These biomarker levels could be altered due to the disease.

Insulin in the brain is associated with learning and memory [42] **.** While peripheral insulin could reach the brain via insulin transporters at the blood brain barrier, statin could affect this process [43], [44]. Prolonged periods of increased peripheral insulin have the opposite effect of down regulating the transport of insulin across into the brain [45]. This suggests that diabetic subjects with statin treatment could have altered brain insulin level and affect cognitive performance. Furthermore, this observation is also in line with our analysis that higher levels of insulin resistance are correlated to poorer attention. According to Mapou's framework for assessment [46], attention and executive function abilities are considered as fundamental to effective expression of other abilities such as learning and memory.

However, it should be noted that there are limitations to the interpretation of the data from this study. This is a pilot study with relatively small sample size, so detecting cognitive deficits in a larger group of diabetics cannot be ruled out. In addition, the age of the population studied is generally younger with less confounding health issues than that reported in previous studies [8], [9], [29]–[33], [47]. When this population aged, they may be more susceptible to cognitive deficits. Thus a larger study with a more heterogeneous population of diabetics drawn from a clinical setting is required to confirm the current findings.

As this pilot study has only examined male subjects, it is unclear if similar conclusions can be derived from female subjects since the trajectory of diabetes differs between the genders [48].

Higher HbA1c values have been reported to correlate with lower cognitive function [49]. However, this biomarker was not measured during the study. Therefore, no correlation analysis was conducted with HbA1c. It is possible that the subjects in this study have good glycemic control, which would be less likely to affect cognitive function.

Although correlations were seen between biochemical markers and cognitive domains, they were restricted to diabetics on statins, no correlations were seen in diabetics not on statins.

In conclusion, there was no evidence of cognitive impairment in this local Chinese male diabetic cohort studied. Further, statin usage in this male diabetic subject cohort did not affect their cognitive performance as compared to diabetic non-statin users. However, diabetic statin users in this cohort may be susceptible to dysfunction in the domains of attention and executive function. Peripheral biomarkers may be used to predict declining cognitive performance.

## Supporting Information

Table S1 Table shows performance (*composite scores based on z-scores*) on cognitive domains of modified Harmonization protocol after controlling for age, education, BMI and duration of diabetes using ANCOVA.
(DOCX)

Table S2 Table shows performance (composite scores based on *z­*scores) on cognitive domains of modified Harmonization protocol after controlling for age, education, BMI, duration of diabetes, using ANCOVA.
(DOCX)
