# Supplementary material for: A Pilot Study to Examine the Correlation between Cognition and Blood Biomarkers in a Singapore Chinese Male Cohort with Type 2 Diabetes Mellitus
Source: PLoS One. 2014 May 9;9(5):e96874. doi: 10.1371/journal.pone.0096874 (PMC4016130; doi:10.1371/journal.pone.0096874)
Supplement: Table S1 — Table shows performance (composite scores based on z-scores) on cognitive domains of modified Harmonization protocol after controlling for age, education, BMI and duration of diabetes using ANCOVA. (DOCX) [file pone.0096874.s001.docx]

Table S1. Table shows performance (*composite scores based on z-scores*) on cognitive domains of modified Harmonization protocol after controlling for age, education, BMI and duration of diabetes using ANCOVA.


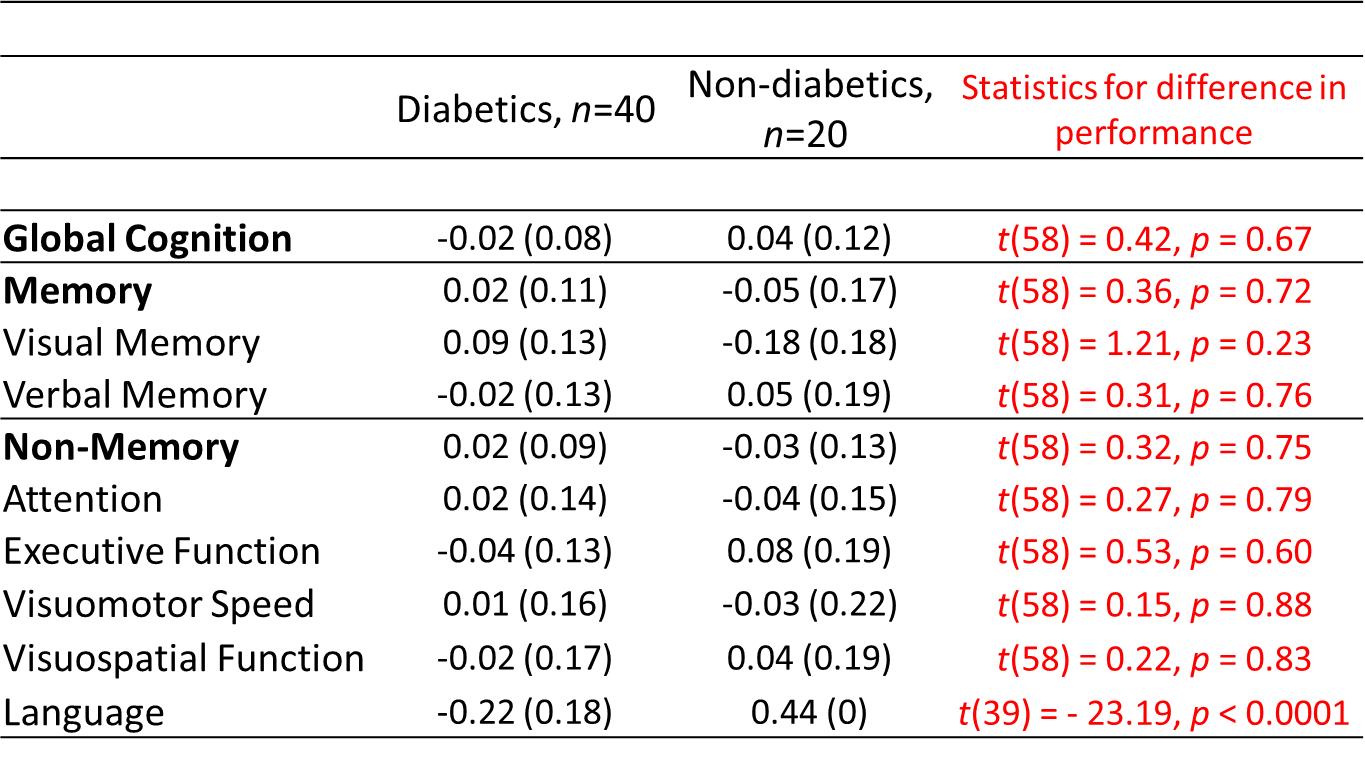


Note: Standard error in parentheses.
