# Supplementary material for: A Pilot Study to Examine the Correlation between Cognition and Blood Biomarkers in a Singapore Chinese Male Cohort with Type 2 Diabetes Mellitus
Source: PLoS One. 2014 May 9;9(5):e96874. doi: 10.1371/journal.pone.0096874 (PMC4016130; doi:10.1371/journal.pone.0096874)
Supplement: Table S2 — Table shows performance (composite scores based on z­scores) on cognitive domains of modified Harmonization protocol after controlling for age, education, BMI, duration of diabetes, using ANCOVA. (DOCX) [file pone.0096874.s002.docx]

Table S2: Table shows performance (composite scores based on *z­*scores) on cognitive domains of modified Harmonization protocol after controlling for age, education, BMI, duration of diabetes, using ANCOVA.

|  | | | |
| --- | --- | --- | --- |
|  | Statin Users,  *n*=22 | Non-statin Users, *n*=18 | Statistics for difference in performance |
| **Global Cognition** | -0.02 (0.11) | -0.03 (0.12) | *t*(38) = 0.06, *p* = 0.95 |
| **Memory** | 0.05 (0.14) | -0.01 (0.18) | *t*(38) = 0.27, *p* = 0.79 |
| Visual Memory | 0.12 (0.17) | 0.01 (0.19) | *t*(38) = 0.06, *p* = 0.95 |
| Verbal Memory | 0.04 (0.16) | -0.11 (0.22) | *t*(38) = 0.56, *p* = 0.58 |
| **Non-Memory** | -0.00 (0.12) | 0.02 (0.13) | *t*(38) = 0.11, *p* = 0.91 |
| Attention | 0.12 (0.20) | -0.10 (0.19) | *t*(38) = 0.79, *p* = 0.44 |
| Executive | 0.10 (0.21) | -0.19 (0.19) | *t*(38) = 1.00, *p* = 0.32 |
| Visuomotor Speed | -0.08 (0.23) | 0.10 (0.15) | *t*(38) = 0.62, *p* = 0.54 |
| Visuospatial Function | 0.13 (0.14) | -0.20 (0.34) | *t*(38) = 0.96, *p* = 0.34 |
| Language | -0.16 (0.24) | -0.30 (0.29) | *t*(38) = 0.38, *p* = 0.71 |

Note: Standard Error in parentheses.
